# Vitamin D Deficiency and Insufficiency According to the Current Criteria for Children: Vitamin D Status of Elementary School Children in Turkey

**DOI:** 10.4274/jcrpe.galenos.2018.2018.0272

**Published:** 2019-05-28

**Authors:** F. Sinem Hocaoğlu-Emre, Devrim Sarıbal, Osman Oğuz

**Affiliations:** 1 Beykent University School of Health Sciences, Department of Nutrition and Dietetics, İstanbul, Turkey; 2 İstanbul University Cerrahpaşa, Cerrahpaşa Faculty of Medicine, Department of Biophysics, İstanbul, Turkey; 3 İstanbul Training and Research Hospital, Department of Clinical Chemistry, İstanbul, Turkey

**Keywords:** 25(OH) vitamin D, vitamin D deficiency, vitamin D insufficiency, primary school children, vitamin D levels

## Abstract

**Objective:**

This study aimed to determine the ratio of seasonal vitamin D deficiency and insufficiency in elementary school children aged between 6-9 years old, living in one of the largest metropols of Europe, İstanbul.

**Methods:**

Serum 25(OH)D levels of 640 children aged 6-9 years old were scanned retrospectively from the hospital information system records between September 2017-August 2018 period. Vitamin D deficiency was defined as a serum 25(OH)D level less than 12 ng/mL (30 nmol/L) and insufficiency as levels between 12 and 20 ng/mL (30-50 nmol/L).

**Results:**

Serum 25(OH)D levels ranged from 3.90 to 64.60 ng/mL, the median value was 25.95 ng/mL for all subjects. Of all the primary school children, 485 (75.78%) had adequate levels of 25(OH)D. Vitamin D deficiency was observed in 36 of children (5.62%), whereas insufficient levels of 25(OH)D were found in 119 children (18.60%). The ratio of vitamin D insufficiency and deficiency together was highest in spring (31.87%) and lowest in summer (13.12%).

**Conclusion:**

Vitamin D deficiency is a widely observed and preventable public health problem among children of different ages. It is necessary to increase the awareness among health professionals, and providing 25(OH)D supplements will yield generations with healthy bone structure and well growth.

**What is already known on this topic?**

Vitamin D deficiency and insufficiency is a widely observed condition among children, especially in winter and post-winter periods.

**What this study adds?**

Our study determined the frequency of vitamin D deficiency and insufficiency in a large group of children of elementary school age, based on seasonality. Particularly, this is the first study, determining serum 25(OH)D levels in this age group, subclassified into different seasons.

## Introduction

25-OH vitamin D [25(OH)D] started to gain importance worlwide for its important role in healthy bone structure and calcium and phosphate metabolism. There are many studies showing 25(OH)D deficiency and insufficiency in children worldwide (1). In the presence of 25(OH)D deficiency and insufficiency, absorbtion of both calcium and phosphorus is impaired resulting in reduced bone mineral density (2).

Low levels of 25(OH)D affects an individual’s present and future health status, triggering multiple systemic responses reducing bone density and the level of immune response since there are 25(OH)D receptors in a wide range of tissues, and are related with retarded growth, skeletal deformities and secondary hyperthyroidism in the childhood, whereas hip fracture in the elderly is observed in individuals with impaired bone structure (3,4,5). Also, there are increasing data explaining the relationship between low levels of 25(OH)D and different types of non-skeletal diseases including some types of cancer, autoimmune, infectious, cardiovascular and psychiatric diseases (6).

Risk factors for 25(OH)D deficiency in children were defined as obesity, intestinal malabsorbtion syndromes, usage of anticonvulsant agents such as Phenytoin, phenobarbital, and carbamazepine, low levels of sun exposure, clothing habits, climatization and seasonality, nutritional choices, dark skin color (5).

In order to determine an individual’s vitamin D status, serum 25(OH)D level is measured. There are different threshold points used to determine 25(OH)D status of individuals as suggested by different organizations and in guidelines (7,8). Regular measurement of 25(OH)D levels in the childhood and replace the low levels with vitamin D fortification or supplementation is essential and a public health matter in order to acquire healthy generations with robust bone structure.

The aim of our study was to assess serum 25(OH)D levels in elementary school children aged between 6-9 years old within a year duration and determine 25(OH)D status between different seasons.

## Methods

### Study Population and Analysis

This is a retrospective study, conducted in one of the largest training hospitals in İstanbul, Turkey. Between September 2017-August 2018, children aged between 6-9 years old who underwent annual check-up for vitamin D status were randomly selected according to the block randomization method in order to create sampling groups of equal sample sizes from hospital information system. All of the participants in our study resided in a large metropolitan area. All were Caucasian and of Turkish origin. Health status of children were controlled from their medical data and children who have a chronic disease, eg. diabetes, inflammatory bowel disease, obesity were excluded. Additionally, children with extremely high and toxic levels of 25(OH)D were excluded, assuming the use of vitamin D supplementation. Of these children fitting to our inclusion criteria, 160 children (80 female, 80 male) were randomly selected from the hospital records for each age group in terms of their sampling and analysis date. One hundred sixty children from each age group were then divided into subgroups of 40 subjects (20 female, 20 male) for each seasons. Seasons in our climate zone are as follows: Fall (September to November), Winter (December to February), Spring (March to May) and Summer (June to August).

The study was approved by the Institutional Ethical Committee of İstanbul Training and Research Hospital (no. 2018/1499).

Serum 25(OH)D levels were measured with chemiluminescence method using Access 25(OH) vitamin D total test (Beckmann Coulter, Inc., USA). Inter-assay and intra-assay CV% as supplied by the manufacturer were between 5.1-8.1% and 2.2-4.7%, respectively. The analysis laboratory was a participant of the RIQAS Immunoassay Speciality-1 External Quality Assurance program (Randox Laboratorıes Ltd., United Kingdom) for 25(OH)D and no deviation in the internal and external quality control results were observed within the study duration.

### Determination of Vitamin D Status

Different classification algorithms were recruited for the determination of vitamin D status in the literature, so far. In our study, we used the widely used cut-off points as suggested by Munns et al (8). According to their criteria, vitamin D deficiency was defined as a serum 25(OH)D level of <12 ng/mL (<30 nmol/L) and insufficiency as a 25(OH)D level between 12 and 20 ng/mL (30-50 nmol/L). 25(OH)D levels higher than 20 ng/mL (50 nmol/L) was accepted as adequate.

### Statistical Analysis

Data are expressed as mean ± standard deviation. In the tables, the lowest and highest values were defined as well as the medians. The rate of deficiency, insufficiency and adequacy were shown as the number and percentage of the cases within eacy subgroup. Statistical analyses were done using the SPSS for windows (SPSS Inc, Chicago, IL). Comparisons of means between two groups were done using Student’s t-tests. The ratios of vitamin D deficiency between groups were compared using the χ^2^ test. The results were evaluated using a significance value of p<0.05.

## Results

Table 1 presents the mean serum 25(OH)D concentrations among different age groups of the study population. According to these measurements, we could not find a statistically significant difference between different genders of children in same age groups. When all age groups were compared in terms of gender, and independent of the seasonality, we could not find a difference between boys and girls. When all 640 children were evaluated together, serum 25(OH)D levels ranged from 3.90 to 64.60 ng/mL; the median value was 25.95 ng/mL. Division of children into subgroups in terms of age, gender, and seasonality did not yield a statistically significant difference between boys and girls of the same group (Table 2).

**Figure 1:**
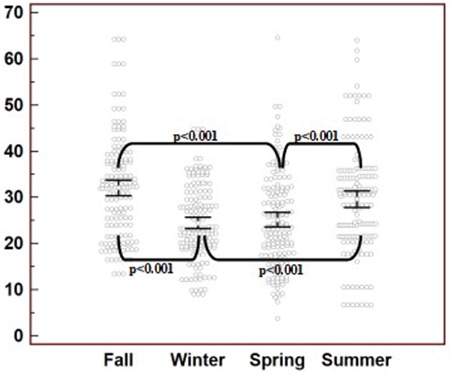
Distribution of 25(OH)D in different seasons. The y axis represents the 25(OH)D levels and their seasonal distribution. Significant differences were depicted with conjuncted lines between the seasons using p values

Mean 25(OH)D levels of all subjects were found to be 32.11±11.24 in fall, 24.24±7.95 in winter, 25.18±10.09 in spring and 29.69±11.53 in summer seasons. When mean levels of 25(OH)D were compared between seasons, levels measured in winter and spring were significantly lower than the levels in summer and fall (p<0.001) (Figure 1).

Analysis of the vitamin D status in terms of age and gender was shown in Table 3. Of all the 640 primary school children, 485 (75.78%) had adequate levels of 25(OH)D. Vitamin D deficiency was observed in 36 of children (5.62%), whereas insufficient levels of 25(OH)D were detected in 119 children (18.60%). According to our data, the highest rates of deficiency and insufficiency were found in the 8-year old children’s group (8.12%; 21.25%, respectively). Furthermore, when the children were divided into subgroups in terms of age and gender, we detected a statistically significant difference between 6-year old girls and boys for the rate of deficiency, and 7-year old girls and boys for the rate of insufficiency (Table 3).

**Figure 2:**
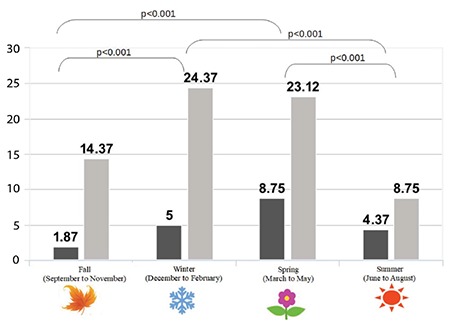
Rate of vitamin D deficiency and insufficiency by season. 25(OH)D <20 ng/mL accepted as deficiency (black color); 25(OH)D between 21-29 ng/mL accepted as insufficiency (grey color). Significant differences were depicted with conjuncted lines between the seasons using p values

The rate of vitamin D insufficiency and deficiency together was highest in spring, and lowest in summer season (13.12%) (Table 4). Comparison of insufficiency and deficiency rates between the seasons fall-winter, winter-summer, spring-summer, fall-spring yielded statistical significance (Figure 2).

## Discussion

Our study includes Turkish elementary school children of both genders between 6-9 years old, residing in the largest metropol of Turkey, and one of the largest metropols of Europe, İstanbul. The subjects were chosen among the healthy children, who underwent laboratory tests for their routine annual check-up.

There are different suggested cut-off points for evaluation of vitamin D status. The Endocrine Society accepts a threshold of 12-20 ng/mL for insufficiency, and >20 ng/mL to represent sufficiency (8). However, the Institutes of Medicine claims that 25(OH)D levels above 20 ng/mL does not supply an additional benefit for bone health (7). Based on these approaches, the prevalence of insufficient and deficient individuals highly vary between the studies worldwide. In our study, we used the cut-off points determined by the Endocrine Society, since that classification would be most appropriate for our group.

Our study demonstrated that, taken together, a total of 155 children out of 640 (24.21%) had deficient and insufficient levels of 25(OH)D in their blood serum. We also found that, the 25(OH)D levels were significantly differed depending on the seasonality. The rate of deficient and insufficient levels of 25(OH)D were higher in the winter and spring, when compared to other seasons. Additionally, while we compared the number of children with inadequate levels of 25(OH)D, we found that 78 (12.18%) of were girls, whereas 77 (12.03%) of were boys.

This is the first and wide analysis of vitamin D status among primary school age group of children in Turkey. There is a range of studies showing deficient or inadequate levels of 25(OH)D in children, worldwide. The findings of our study have similar and different findings with other studies of Turkish and European origin.

In their study with Turkish children of 11-18 ages, Karagüzel et al (9) used the cut-off value 20 ng/mL for deficiency, and found the prevalence of vitamin D deficiency 93% during spring and 71% during autumn seasons, with an overall prevalence of 82%. While we used cut-off point 12 ng/mL for deficiency in our study group, we found vitamin D deficiency was 8.75% during spring, and 1.87% during autumn. Additionally, insufficient levels of vitamin D were detected in the spring and autumn seasons, with a rate of 23.12% and 14.37%, respectively. The difference between their study and ours might be due to the following reasons: Firstly, their study group consisted of a different age group than our group, who are teenagers, among which are girls wearing traditional clothing covering their body as a result of regional beliefs. Secondly, their study was conducted in the northeastern part of Turkey with a colder and less sunny seasonal times when compared to İstanbul (10). Lastly, their cut-off points are higher than the values we recruited for the classification of our subjects.

Erol et al (11) also measured the 25(OH)D levels of 280 children aged 3-17 years old, living in İstanbul, Turkey, the same region our study group is located. They used the classification suggested by American Pediatric Endocrine Association defining a serum 25(OH)D level less than 15 ng/mL as deficiency, and levels between 15 and 20 ng/mL as insufficiency. Of the individuals, they found 80.36% rate of deficiency and 11.79% rate of insufficiency in the end of winter samples.

In a study from Kuwait, recruiting the similar age group subjects, the defined that being ≤8.5 years old is a significant risk factor for vitamin D deficiency (12). This finding is consistent with our data showing higher rate of deficiency and insufficiency in 8 years old children. The similarity between two studies might be a result of accelerated growth in children of this age.

In their study analyzing vitamin D status’ of a large group of Greek children between 9-13 years old, and using the same cut-off points with our study, Manios et al found that the overall prevalence of vitamin D deficiency and insufficiency were 5.2% and 52.5%, respectively (13). The lower rate of vitamin D deficient children in our study group might be a result of their sampled age group and this group’s increased demand to 25(OH)D as a result of accelerated growth. Additionally, they did not include the samples on the summer season, since they collected samples from schools, and the schools were on summer break from June to September. They also observed a higher prevalence of low 25(OH)D levels in girls, when compared to boys. Female gender is a well-known reason of both vitamin D deficiency and insufficiency, and data discussing this issue suggested different reasons. Traditional clothing as a result of religional beliefs is one of the arguments that is put forward (14). However, 25(OH)D levels were still found to be lower in girls residing in countries that traditional clothing is not widely used (13,15,16). Studies collecting dietary 25(OH)D intake of children revealed that mean intake of 25(OH)D with food consumption is lower in girls when compared to boys of same age (17). Additionally, tendency to outdoor activities and time spent under direct sunlight is lower in girls (9,11). Recently, female sex hormones (mainly estradiol and estrogens) were shown to be affecting the 25(OH)D levels in females altering the synhesis and metabolism (18).

With the increased level of knowledge and awareness on vitamin D and its relation to growth and disease susceptibility, 25(OH)D fortified foods were started to be sold in public markets in some countries (19). In Turkey, 25(OH)D fortified foods are not common in markets and their prices are higher when compared to the similar group of foods. Thus, for the individuals with low income, vitamin D synthesis through proper and adequate sun exposure remains the sole choice. It has been suggested that sunlight exposure of dorsal body areas for 15 minutes at least three times a week is sufficient to maintain adequate levels of 25(OH)D for adults. In case of diminished or decreased sun exposure due to different reasons including low level of outdoor activities, clothing habits, climate changes, 25(OH)D supplementation is required if the consumed amount of 25(OH)D with foods is not sufficient (1). There are data presenting higher mean blood 25(OH)D levels and lower prevalence of insufficiency and deficiency in children from colder regions of world, suggesting that the higher rate of consumption of fish oil and fish types living in cold sea habitats helped those children to maintain the blood 25(OH)D levels within adequate limits, despite their less exposure to sunlight when compared to the children of warm regions (20,21).

A five-year nationwide ‘vitamin D prophylaxis augmentation programme’ was initiated in 2005 with a collaboration between Turkish Pediatric Endocrine Society and Ministry of Health of Turkey, recruiting free distribution of vitamin D drops to all 0-12 months old children. Consequently, these efforts resulted in a decline in the prevalence of rickets from 6% in 1998 to 0.1% in 2008 in children under 3 years of age (22). However, our study and other forementioned studies reveal the need for vitamin D supplementation for children of different age groups. Although vitamin D supplements are sold for very low prices, and the drop, powder, ampoule forms are covered by government insurance and can be obtained free, there is still need for public awareness in order to provide adequate levels of 25(OH)D for children. Since the administration of supplements to children is under the control of their parents, parent integration is highly essential even though medical and legal authorities provide the sufficient support.

### Study Limitations

Our study has several limitations. Since we used a retrospective data obtained from hospital records of our study group, there is lack of data regarding BMI, social status, time spent under daylight, parathormone levels, dressing habits and daily 25(OH)D intake of children. Additionally, this is a one center study held in İstanbul, thus it does not reflect the status of all Turkish children.

One of the strengths of our study is that it is distinctive for comprising large number of healthy elementary school children aged between 6-9 years and grouping them according to gender and age.

## Conclusion

Our findings reveal that vitamin D deficiency and insufficiency is a common condition on winter and spring times, among children of elementary school age. 25(OH)D supplementation and close follow-up of vitamin D status especially in the winter and post-winter period are required to supply a strong bone structure and healthy growth. Children with adequate levels of 25(OH)D and healthy skeleton will benefit in their adult years, thus this is a public health issue, and should be taken into consideration by authorities using the recommendations from appropriate guidelines.

## Figures and Tables

**Table 1:**
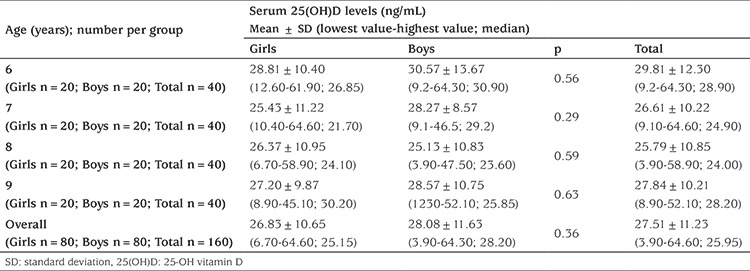
25(OH)D levels of children for different age groups

**Table 2:**
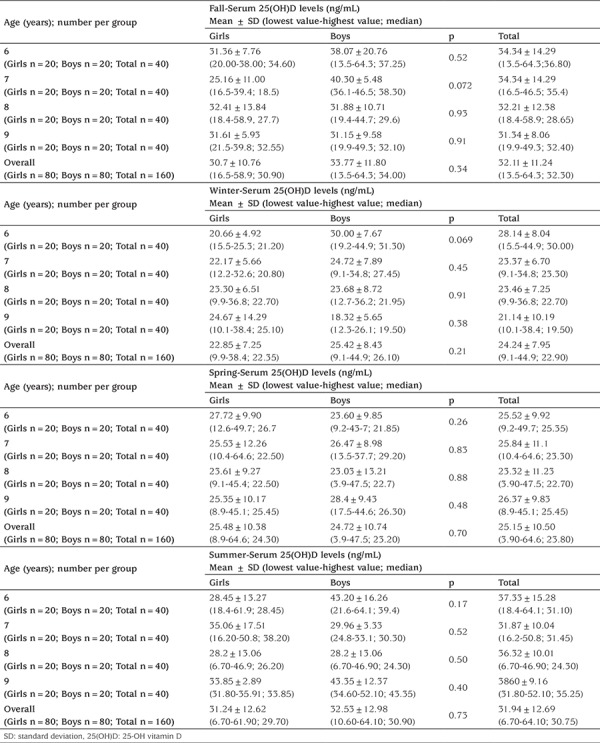
25(OH)D levels of children in different age groups subdivided into seasonal categories

**Table 3:**
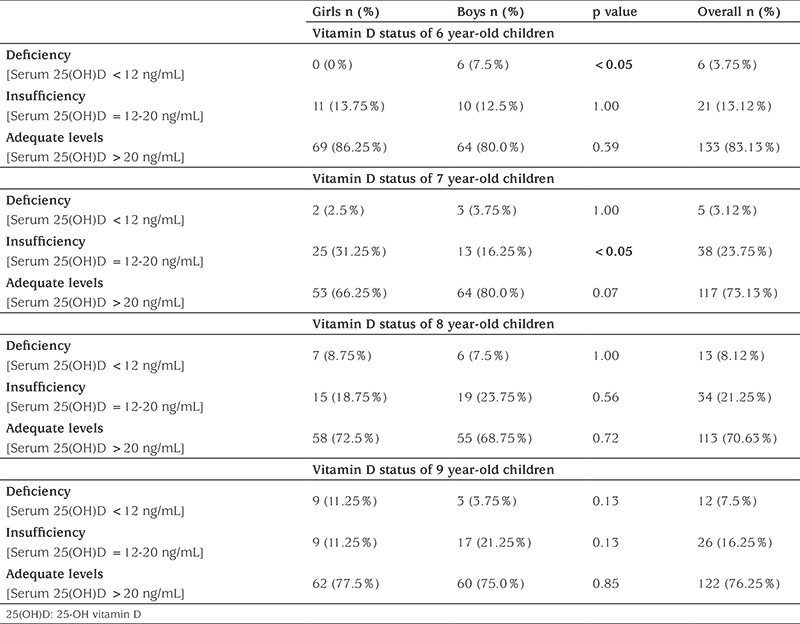
The rate of vitamin D deficiency and insufficiency according to age and gender

**Table 4:**
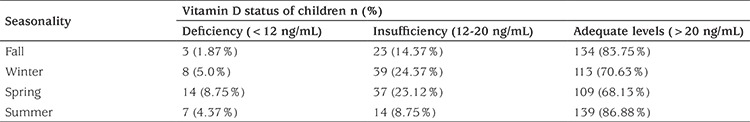
The rate of overall vitamin D deficiency and insufficiency in different seasons
